# Mapping the physical topology of the tumor immune microenvironment: macroscopic image fractals as surrogate markers for immune exclusion phenotype and treatment resistance in colorectal cancer liver metastases

**DOI:** 10.3389/fimmu.2026.1889571

**Published:** 2026-09-17

**Authors:** Guo Ziyi, Wentao Zhou, Kailin Zou

**Affiliations:** 1 Department of Nuclear Medicine, Jinzhou Medical University, Jinzhou, China; 2 Department of Nuclear Medicine, First Affiliated Hospital of Jinzhou Medical University, Jinzhou, China; 3 Department of Nuclear Medicine, The Third Affiliated Hospital of Jinzhou Medical University, Jinzhou, China

**Keywords:** colorectal cancer liver metastases, explainable artificial intelligence, fractal dimension, immune exclusion phenotype, treatment resistance, tumor immune microenvironment

## Abstract

**Background:**

The spatial tumor immune microenvironment (TIME) determines therapy response in colorectal cancer liver metastases (CRLM). To overcome biopsy sampling bias, this study investigates multimodal macroscopic image fractal dynamics to non-invasively map immune physical barriers and metabolic states, constructing a multi-scale immune-radiomics joint score (IRJS) for robust prognostic stratification and exploratory assessment of treatment resistance.

**Materials and methods:**

This dual-center observational study enrolled 270 CRLM patients with dual-modality imaging (contrast-enhanced magnetic resonance imaging, CE-MRI, 18F-fluorodeoxyglucose (FDG) positron emission tomography-computed-tomography, ^18^F-FDG PET/CT) and digital spatial pathology. Patients were spatiotemporally divided into training (n=150), prospective temporal validation (n=45), and external validation (n=75) cohorts. Structural (FD_MRI) and metabolic (FD_PET) fractal dimensions were combined with the systemic immune-inflammation index (SII) and *KRAS* status to build the IRJS using XGBoost, utilizing the SHAP framework to evaluate mapping mechanisms.

**Results:**

FD_MRI positively correlated with α-SMA^+^ pro-fibrotic stromal density (*r* = 0.684), which may reflect the mechanical barrier excluding CD8^+^ T cells. FD_PET showed a significant association with core hypoxia (HIF-1α) and systemic immune exhaustion. IRJS robustly identified the immune-inflamed phenotype across training, temporal, and external validation cohorts (AUCs: 0.921, 0.886, 0.862, respectively). The IRJS-identified inflamed phenotype was an independent protective factor for overall survival (HR = 0.31, *P* < 0.001). Furthermore, IRJS demonstrated significant clinical net reclassification improvement (NRI = 0.384, *P* < 0.001) and net clinical benefit in decision curve analysis.

**Conclusion:**

Dual-modal fractal dynamics provide non-invasive surrogate markers for mechanical constraints and metabolic exhaustion in CRLM. The IRJS model enables robust spatiotemporal assessment of immune evasion, offering multidisciplinary teams a potential framework to explore non-invasive biopsy decisions, prognostic stratification, and provide exploratory insights into potential immunotherapy resistance.

## 1 Introduction

Colorectal cancer (CRC) ranks third in global incidence and second in mortality, with mortality primarily driven by the occurrence of liver metastases (CRLM) (1). While progress has been made in systemic therapies, the heterogeneity of the tumor immune microenvironment (TIME) within CRLM remains a limiting factor for precision therapy (2). The TIME operates as a highly complex and dynamic ecosystem—composed of malignant clones, infiltrating immune cells, stromal cells, extracellular matrix (ECM), and soluble factors—that collectively dictate tumor progression, immune escape, and therapeutic response (3). Research indicates that the spatial distribution pattern of CD8^+^ cytotoxic T cells (immune-inflamed, immune-excluded, and immune-desert phenotypes) determines patient clinical response to chemotherapy, targeted therapy, and immune checkpoint blockade (ICB) (4, 5).

Defining these spatial immune phenotypes in clinical practice presents challenges (6). Although microsatellite instability-high (MSI-H) accounts for a small fraction of advanced CRLM, most microsatellite stable (MSS) patients harbor intratumoral immune heterogeneity, which conventional genetic sequencing cannot fully resolve (7). Traditional histopathology is limited by invasiveness and spatial sampling bias, making it difficult to capture the tumor landscape comprehensively. Artificial intelligence (AI) based on medical imaging provides a non-invasive assessment method; however, existing high-dimensional radiomics models (e.g., those relying on linear texture features based on gray-level co-occurrence matrices, GLCM) often lack mechanistic explanation (8). These features, based on statistical distributions, often lack biological context and do not fully account for the spatial polarization of immune cells or the cross-scale communication between local microenvironment heterogeneity and the host systemic immune network (9).

Addressing this methodological limitation requires considering the biophysical properties of the tumor microenvironment (10). Tumors are nonlinear dynamic systems driven by angiogenesis, matrix remodeling, and clonal evolution (11). Traditional methods for assessing microenvironment heterogeneity rely on conventional radiomics, but their linear statistical features based on Euclidean geometry presume relatively regular tumor boundaries and homogeneous density. This makes them susceptible to equipment batch effects and limits their utility in clinical decision-making due to a lack of biophysical interpretation (12). Fractal geometry, using the fractal dimension (FD), can quantify irregular structures within the microenvironment (13). In this study, we propose that multimodal imaging fractals can serve as cross-scale surrogate markers for microscopic physical and metabolic states, based on the following mechanisms:

Structural Fractal (FD_MRI) as a Potential Surrogate for Mechanical Physical Barrier: High FD_MRI is hypothesized to correlate with the tortuosity of the neo-vasculature and the disordered deposition of the extracellular matrix (ECM) mediated by fibroblast activation. This local physical state constitutes an *in vivo* surrogate marker for elevated interstitial fluid pressure (IFP) and shear stiffening, which we propose maps the physical constraint barrier that traps and excludes T cells (14).

Metabolic Fractal (FD_PET) Mapping Metabolic Survival Competition: In standard clinical settings, ^18^F-FDG PET/CT is used for systemic macroscopic assessment and identifying primary/metastatic lesions, while high-resolution contrast-enhanced magnetic resonance imaging (CE-MRI) is used for detailed structural characterization of liver lesions. Structural fractal (FD_MRI) utilizes high-frequency MRI to map the mechanical physical barrier, while metabolic fractal (FD_PET) uses systemic metabolic mapping to reflect local metabolic competition. Indeed, the integration of nuclear medicine with other high-resolution radiological modalities represents a highly promising future prospect for comprehensively decoding tumor heterogeneity and developing precision companion diagnostics (15). FD_PET characterizes the nonlinear heterogeneity of anaerobic glycolysis metabolism (Warburg effect). This metabolic profile maps the hypoxic state (HIF-1α aggregation) and accumulation of acidic lactate in the tumor core. This metabolic environment impairs effector T cell function through substrate deprivation and disrupts metabolic signaling axes, driving the polarization of infiltrating T cells towards an exhausted phenotype and promoting an immune-desert microenvironment (16).

Based on this framework, we propose a cross-scale tumor physical-immune coupling hypothesis: Secretion of pro-fibrotic cytokines (e.g., TGF-*β*) mediated by underlying oncogenic drivers (e.g., *KRAS* mutations) correlates with fibroblast activation and vascular remodeling within the microenvironment. These microscopic events induce macroscopic geometric changes, which construct a mechanical constraint barrier hindering T cell infiltration and act as an immune sink. By releasing metabolic waste and pro-inflammatory cytokines, this process triggers systemic immune exhaustion in the host.

To evaluate this hypothesis, this study conducted a large-sample clinical investigation encompassing two centers, including a temporal validation cohort. The study aims to non-invasively map physical and metabolic states in local CRLM using multimodal imaging fractal dimensions, integrated with systemic immune-inflammation indices (SII, NLR, LDH) as macroscopic readouts of the systemic immune sink effect. This integration constructs an explainable machine learning model (IRJS). Using SHAP analysis, we evaluate the causal regulatory network among macroscopic fractal readouts, microscopic physical barriers, and systemic immune exhaustion, providing a companion diagnostic approach for CRLM patients based on biophysical mechanisms.

## 2 Materials and methods

### 2.1 Study design and participants

This study is a multicenter, retrospective, and temporally split-validation observational clinical study (as illustrated in the comprehensive screening and technical roadmap flowchart shown in **Figure 1**). The study protocol adhered to the Strengthening the Reporting of Observational Studies in Epidemiology (STROBE) statement (17) and the Transparent Reporting of a Multivariable Prediction Model for Individual Prognosis or Diagnosis (TRIPOD) guidelines (18). Patients treated between March 1, 2019, and December 31, 2025, were consecutively screened. The study protocol strictly adhered to the ethical principles of the Declaration of Helsinki and received formal approvals from the Independent Clinical Research Ethics Committee of the First Affiliated Hospital of Jinzhou Medical University (Approval No: 2026LL-KY-063) (Center A) and Third Affiliated Hospital of Jinzhou Medical University (Center B). For the retrospective historical cohort (2019–2024), the ethics committee waived the requirement for informed consent. The independent validation cohort for 2025 and the platelet-poor plasma (PPP) samples for cytokine detection were obtained from the hospital’s prospectively constructed standardized tumor biobank; all patients provided written informed consent before enrollment and blood collection.

**Figure 1:**
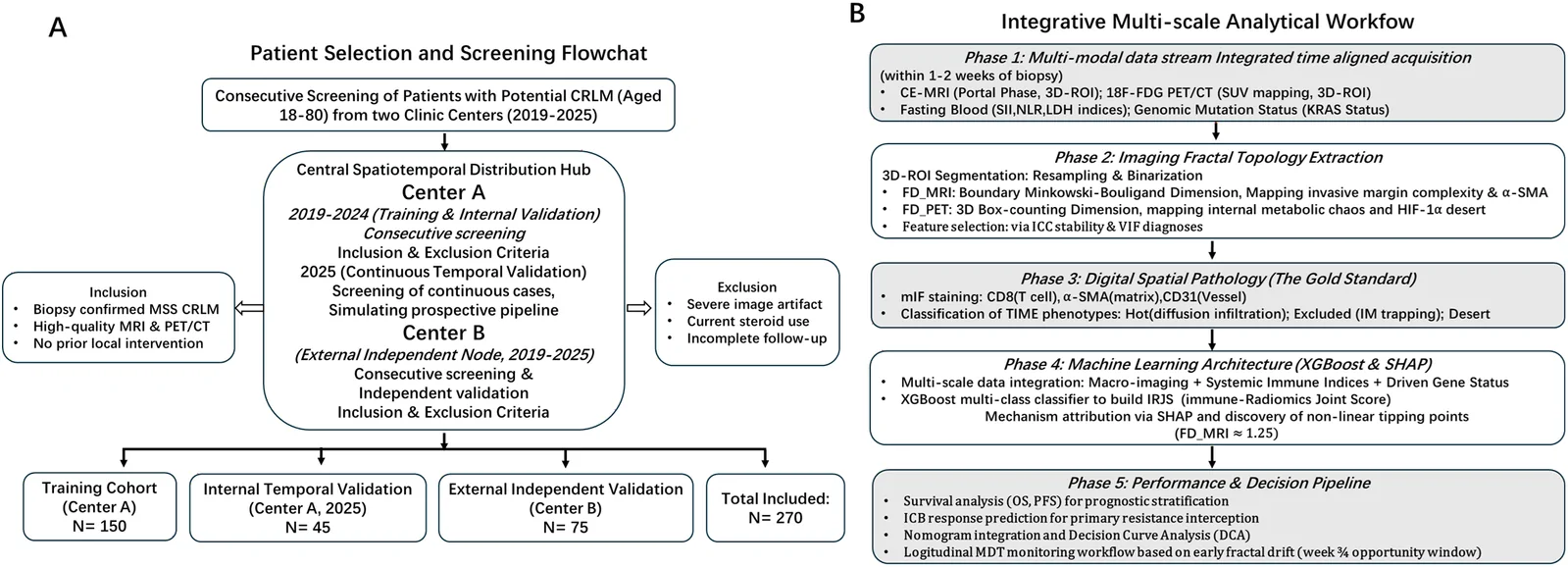
Patient screening flowchart and cross-scale analysis workflow for non-invasive decoding of spatial immune landscapes in CRLM. **(A)** Spatiotemporal screening detailing the consecutive patient enrollment across two clinical centers (2019–2025). The total cohort (N = 270) was allocated into a training/internal validation cohort (Center A, 2019–2024, N = 150), an internal temporal validation cohort (Center A, 2025, N = 45), and an external independent validation cohort (Center B, N = 75). Inclusion and exclusion criteria are specified. **(B)** The multi-scale analysis workflow outlines a five-stage pipeline: (1) acquisition of spatiotemporally aligned multimodal data; (2) independent extraction of macroscopic imaging fractal topologies, using the Minkowski-Bouligand dimension for MRI (FD_MRI) to map stromal barriers, and the 3D box-counting dimension for PET (FD_PET) to map metabolic chaos; (3) establishment of the digital spatial pathology gold standard via multiplex immunofluorescence (mIF); (4) construction of the immune-radiomics joint score (IRJS) using an XGBoost architecture, with SHAP applied to identify non-linear mechanistic thresholds; and (5) evaluation of prognostic performance and treatment response to guide longitudinal monitoring by multidisciplinary teams (MDT).

#### 2.1.1 Inclusion criteria

1. Confirmed clinical and histopathological diagnosis of colorectal cancer liver metastases (CRLM), with the primary tumor resected or diagnosed via endoscopy. Within the total enrolled cohort (n=270), 205 patients (75.9%) provided definitive surgical resection specimens, serving as a robust spatial reference, while 65 patients (24.1%) provided core needle biopsy (CNB) specimens;
2. Availability of baseline CE-MRI, including portal venous phase and ^18^F-FDG PET/CT images before liver lesion tissue sampling (surgical resection or core needle biopsy), with the median interval between imaging and pathological biopsy controlled within 7 days;
3. Complete peripheral blood routine and biochemical test data within 2 weeks before lesion biopsy;
4. Molecular testing reports for core driver genes;
5. Sufficient retained tissue sample volume for subsequent spatial immunohistochemistry (IHC) analysis.

#### 2.1.2 Exclusion criteria

1. Local interventional procedures (e.g., radiofrequency ablation, transarterial chemoembolization) performed on the target lesion before imaging;
2. Artifacts in raw images;
3. Concurrent acute severe infection, active autoimmune disease, or administration of systemic glucocorticoids or non-specific immunomodulatory drugs within 2 weeks before baseline blood draw, to minimize confounding noise from non-tumor systemic inflammation on baseline measurements of serological systemic immune indices (SII, NLR).

#### 2.1.3 Treatment homogeneity

Enrolled patients received first-line systemic therapy according to concurrent NCCN and CSCO clinical practice guidelines to minimize the interference of treatment heterogeneity on survival analyses.

#### 2.1.4 Cohort allocation

Patients treated at Center A from January 2019 to December 2024 were defined as the Discovery and Training Cohort. Patients treated at Center A from January 2025 to December 2025 were designated as the Internal Prospective Temporal Validation Cohort. Patients from Center B (January 2019 – December 2025) were allocated as the External Independent Validation Cohort. Center B used medical imaging hardware (3.0T MRI scanner, Signa HDx, GE, USA) and PET/CT acquisition/reconstruction protocols that differed from Center A, to test the generalization ability of the IRJS model.

### 2.2 Study endpoints

#### 2.2.1 Primary endpoint:

To evaluate the diagnostic performance of the multi-scale XGBoost joint model (IRJS) in distinguishing the immune-inflamed phenotype from the non-immune-inflamed (excluded and desert) phenotypes in the validation cohorts, represented by the area under the receiver operating characteristic curve (AUC).

#### 2.2.2 Secondary endpoints

1. Overall Survival (OS): Time from baseline imaging examination to death from any cause;
2. Progression-Free Survival (PFS): Time from the start of systemic therapy to imaging-documented disease progression or death;
3. Objective Response Rate (ORR): Proportion of patients achieving complete response (CR) or partial response (PR) after receiving systemic therapy. For patients receiving conventional chemotherapy and targeted therapy, progression and response adhere to RECIST 1.1 criteria. For patients receiving immune checkpoint blockade (ICB)-containing regimens, clinical imaging assessment references the immune-related Response Evaluation Criteria in Solid Tumors (iRECIST) to identify immune therapy-induced pseudoprogression;
4. Incremental Clinical Value: Quantitative evaluation of the net reclassification improvement (NRI) achieved by the IRJS score compared to traditional clinicopathological and genetic staging systems.

### 2.3 Immunological and pathological indicators

A blinded independent central review (BICR) mechanism was employed. Two digestive system pathologists with over 10 years of experience performed histological assessments blinded to patient clinical outcomes and imaging features. In case of disagreement, a third expert rendered arbitration.

#### 2.3.1 Spatial immune phenotype

Quantitative assessment of the density and spatial aggregation of CD8^+^ cytotoxic T lymphocytes in the tumor core (TC) and invasive margin (IM) using IHC. Phenotypes were classified as: immune-inflamed (high infiltration in both TC and IM), immune-excluded (high infiltration in IM, lack of significant infiltration in TC), and immune-desert (lack of effective infiltration in both TC and IM regions). For core needle biopsy (CNB) specimens, a biopsy core containing at least 1 mm width of a tumor-liver parenchyma interface was required for IM assessment. For specimens composed entirely of tumor parenchyma, phenotype determination was adjudicated by combining TC region features from multiple core biopsies and pathological consultation. Specimens that could not be accurately assessed were excluded.

#### 2.3.2 Physical barrier and hypoxia assessment

Quantitative staining analysis included α-SMA (fibroblast activation and matrix remodeling), CD31 (microvessel density), and hypoxia/metabolic markers HIF-1α and GLUT1.

#### 2.3.3 Digital pathology quantitative analysis

Multiplex immunohistochemistry (mIF) and conventional IHC stained slides were converted into whole slide images (WSI) using a digital slide scanner (Aperio AT2, Leica Biosystems) and imported into QuPath (version 0.4.3). Using machine learning cell classifiers, tumor parenchyma and stromal regions were identified. For matrix markers (α-SMA, CD31), the percentage of positively stained pixel area relative to the total tumor stromal area (Area %) was used. For hypoxia/metabolic markers (HIF-1α, GLUT1), quantification was performed using the H-score or positive cell nuclear density (cells/mm²).

#### 2.3.4 Centralized pathology review

Tissue blocks or unstained slides from the external validation cohort (Center B) were transported to the pathology laboratory of Center A. Routine H&E and mIF staining for the entire cohort (n = 270) followed a standardized operating procedure (SOP) using the same batch and clone number of primary antibodies.

### 2.4 Immunoserological and genomic indicators systemic immunoserological indicators

Fasting venous blood was collected, and baseline levels were calculated for the systemic immune-inflammation index (SII = platelet count × neutrophil count/lymphocyte count), the neutrophil-to-lymphocyte ratio (NLR), and serum lactate dehydrogenase (LDH).

#### 2.4.1 Genomic and mismatch repair profiling

Next-generation sequencing (NGS, covering *RAS* and *BRAF* mutation panels) was used to determine oncogenic driver status. Mismatch repair deficiency (dMMR) status was assessed via IHC targeting MLH1, PMS2, MSH2, and MSH6. PCR-based microsatellite instability (MSI) amplification was performed for cross-validation when necessary.

### 2.5 Imaging parameter extraction and reproducibility image acquisition and preprocessing

To prevent data drift and harmonize spatial geometry across different institutional scanners, the hardware (3.0 T MRI Scanner, Vero, Siemens, Germany) and core sequence parameters for CE-MRI portal venous phase scanning at Center A were maintained during the study period. For heterogeneous image data originating from different acquisition protocols (e.g., Center B), B-Spline interpolation via SimpleITK was applied for resampling to 1 × 1 × 1 mm³ isotropic voxels prior to feature extraction, ensuring that volumetric and topological calculations were not skewed by native voxel size disparities. Nearest-neighbor interpolation was applied to the 3D-ROI mask, followed by uniform grayscale discretization.

PET/CT imaging was performed on a uMI 780 scanner (United Imaging Healthcare, Shanghai, China). Patients fasted for at least 6 hours, received ^18^F-FDG (3.7–5.55 MBq/kg), and had a 60 ± 10-minute uptake period. Low-dose CT was acquired at 120 kVp. PET data were acquired in 3D mode and reconstructed using the OSEM algorithm with TOF and PSF modeling. 3D regions of interest (ROIs) were delineated using a semi-automatic region-growing algorithm with a contour threshold of 40% of the lesion’s SUVmax. Two nuclear medicine physicians (>5 years experiences), blinded to clinicopathological data, performed slice-by-slice boundary correction. If the 3D Dice similarity coefficient (DSC) between readers was ≥ 0.85, the spatial intersection of the ROIs was used; if DSC < 0.85, a third expert finalized the boundary.

#### 2.5.1 Macroscopic fractal dynamic parameters

A standardized binarization workflow was used within 3D Slicer. The image voxel array was converted to binary space using Otsu’s thresholding. The standard 3D box-counting dimension method extracted the disorder coefficient. For patients with multiple intrahepatic metastases (>3 lesions), the specific liver metastasis localized by preoperative records for core needle biopsy was designated as the target lesion for imaging feature extraction.

Two radiologists performed semi-automatic delineation of 3D-ROIs. To avoid boundary blurring from spatial co-registration of multimodal images, a decoupled extraction strategy was adopted. The radiologists independently performed delineation of target lesions on CE-MRI and PET/CT SUV images. Fractal topological features were extracted separately and fused via machine learning.

#### 2.5.2 Reproducibility assessment (ICC)

Fifty patient samples were extracted from the training set for quality control (**Supplementary Table 1**). Two radiologists performed feature extraction to calculate inter-observer reproducibility. Radiologist A re-extracted features after a one-month interval for intra-observer reproducibility. Feature matrices with ICC > 0.80 were included in modeling.

### 2.6 Statistical analysis and algorithm transparency

Data analysis was executed using R (version 4.2.2) and Python (version 3.9). The IRJS cutoff threshold was derived from the training cohort (2019-2024) and hard-coded for application in the 2025 temporal validation set and external validation set. The sample size adhered to the metric of 10 independent outcome events per predictor variable (EPV > 10). Hypothesis testing was two-sided with *P* < 0.05 considered statistically significant.

#### 2.6.1 Missing data handling

For non-core covariate data with missing rates <5%, Multiple Imputation by Chained Equations (MICE) based on random forest architecture was used.

#### 2.6.2 Machine learning modeling

Initially, 14 candidate clinical, serological, and imaging variables were evaluated for inclusion. The XGBoost algorithm was used to construct the classification model (**Supplementary Table 2**). To prevent high-dimensional overfitting, a strict two-step feature selection strategy was employed. First, imaging features (FD_MRI, FD_PET) were required to meet a reproducibility threshold of Intraclass Correlation Coefficient (ICC) > 0.80 (**Supplementary Table 1**). Second, variables underwent Variance Inflation Factor (VIF) diagnostic control before training (**Supplementary Table 3**). VIF values were strictly controlled within < 3.5 to eliminate multicollinearity. The model used an adaptive weight adjustment strategy (scale_pos_weight) for class imbalance. GridSearchCV combined with 5-fold cross-validation was used for hyperparameter optimization (19). To justify the utilization of a complex ensemble algorithm, standard Multivariable Logistic Regression and L1-penalized LASSO regression were formulated as baseline comparators. Model performances were statistically compared using DeLong’s test to determine if the non-linear mapping capabilities of XGBoost provided significant incremental diagnostic value over conventional linear statistical approaches. Calibration curves were plotted for the Hosmer-Lemeshow goodness-of-fit test. To further rigorously mitigate overfitting and validate the internal stability of the model given the modest cohort size, a 1, 000-resample bootstrap optimism correction was applied to the training cohort to adjust the apparent Area Under the Curve (AUC).

#### 2.6.3 Model explanation

The SHapley Additive exPlanations (SHAP) algorithm framework was applied for attribution analysis (20). SHAP summary plots and local dependence plots were used to analyze interactive threshold effects.

#### 2.6.4 Survival and prognosis analysis

The Kaplan-Meier method plotted time-survival curves, and the Log-rank test was performed. The proportional hazards assumption was verified using Schoenfeld residual tests before multivariate Cox proportional hazards regression.

#### 2.6.5 Incremental value and data sharing

The study utilized the C-index, continuous NRI, and integrated discrimination improvement (IDI) to quantify incremental efficacy. The XGBoost/SHAP machine learning training code and the Python-based fractal feature extraction script will be publicly released on GitHub.

## 3 Results

### 3.1 Continuous screening of multi-center spatiotemporal validation cohorts and establishment of baseline

Following STROBE and TRIPOD guidelines, a study protocol was established encompassing 270 CRLM patients with complete multimodal imaging and spatial pathology immunotyping. Cases were allocated into: a discovery and training cohort (Center A, 2019–2024, n=150), an internal prospective temporal validation cohort (Center A, 2025, n=45), and an external independent multi-center validation cohort (Center B, 2019–2025, n=75).

Baseline demographics and clinicopathological characteristics among the validation cohorts were well-balanced (**Table 1**). Mean age ranged from 61.8 to 63.1 years (*P* = 0.742), male proportion ranged from 57.8% to 61.3% (*P* = 0.815), and the proportion of TNM stage IV patients remained between 78.7% and 80.0% (*P* = 0.912). *KRAS* mutation rates were 45.3%, 42.2%, and 46.7% (*P* = 0.884). *NRAS* mutations, *BRAF V600E* mutations, and MSI-H/dMMR status (6.7%–8.0%) showed no statistically significant differences (*P* > 0.05). Peripheral blood composite indices (median NLR *P* = 0.490; median SII *P* = 0.385; mean LDH *P* = 0.283) and CEA (*P* = 0.671) were consistent across cohorts.

**Table 1:** Baseline clinical, genomic, and multiscale immune characteristics across multicenter cohorts.

Variables	Training cohort (Center A, 2019-2024) n = 150	Temporal validation (Center A, 2025) n = 45	External validation (Center B, 2019-2025) n = 75	P-value
Demographics and clinical characteristics				
Age (years), mean (SD)	62.4 (9.8)	61.8 (10.2)	63.1 (9.5)	0.742
Sex (Male), n (%)	92 (61.3%)	26 (57.8%)	46 (61.3%)	0.815
TNM Stage (IV with liver metastases), n (%)	119 (79.3%)	36 (80.0%)	59 (78.7%)	0.912
Core genomic drivers				
*KRAS* mutant, n (%)	68 (45.3%)	19 (42.2%)	35 (46.7%)	0.884
*NRAS* mutant, n (%)	4 (2.7%)	1 (2.2%)	2 (2.7%)	>0.99
*BRAF V600E* mutant, n (%)	8 (5.3%)	2 (4.4%)	4 (5.3%)	0.958
MSI-H/dMMR, n (%)	12 (8.0%)	3 (6.7%)	6 (8.0%)	0.781
Systemic immune and inflammatory indices				
Neutrophil-to-Lymphocyte Ratio (NLR), median (IQR)	3.4 (2.1–4.8)	3.2 (2.0–4.5)	3.6 (2.3–5.0)	0.490
Systemic Immune-Inflammation Index (SII), median (IQR)	685 (420–950)	650 (410–920)	710 (450–980)	0.385
Lactate Dehydrogenase (LDH, U/L), mean (SD)	235 (45)	228 (42)	242 (48)	0.283
Carcinoembryonic Antigen (CEA, ng/mL), median (IQR)	45.2 (18.5–110.3)	42.8 (15.2–105.4)	48.5 (20.1–115.6)	0.671
Macroscopic fractal dynamics (imaging)				
Structural Fractal Dimension (FD_MRI), mean (SD)	1.25 (0.08)	1.24 (0.07)	1.26 (0.09)	0.355
Metabolic Fractal Dimension (FD_PET), mean (SD)	1.18 (0.06)	1.19 (0.06)	1.18 (0.07)	0.612
Spatial TIME phenotypes (Pathological gold standard)				
- Immune-inflamed, n (%)	48 (32.0%)	16 (35.6%)	23 (30.7%)	0.528
- Immune-excluded, n (%)	72 (48.0%)	20 (44.4%)	37 (49.3%)	
- Immune-desert, n (%)	30 (20.0%)	9 (20.0%)	15 (20.0%)	

MSI-H, microsatellite instability-high; dMMR, deficient mismatch repair; TIME, tumor immune microenvironment.

Data are presented as n (%), mean (SD), or median (IQR) depending on data distribution. The *P*-value indicates the statistical difference among the three cohorts (Training, Temporal Validation, and External Validation), calculated using analysis of variance (*ANOVA*) for normally distributed continuous variables, Kruskal-Wallis test for non-normally distributed continuous variables, and Pearson’s chi-square test or Fisher’s exact test for categorical variables.

The spatial tumor immune microenvironment (TIME) phenotypes reflected by digital pathology showed the immune-excluded phenotype accounted for the highest proportion (44.4%–49.3%), and the immune-desert phenotype remained at approximately 20.0%, with overall distribution demonstrating consistency among cohorts (*P* = 0.528).

### 3.2 Macroscopic fractal dynamics recapitulate microscopic physical barriers and systemic immune exhaustion

Multiplex immunohistochemistry (mIF) and digital spatial pathology confirmed that the invasive margin of immune-excluded CRLM features an α-SMA^+^ pro-fibrotic stromal barrier and a disordered CD31^+^ microvascular network, which acts as a mechanical constraint to T cell infiltration. The core of immune-desert tumors presented with HIF-1α accumulation, indicating a hypoxic microenvironmental state.

Using the 3D Slicer platform to calculate macroscopic fractal dimensions (FD), structural fractal dimension (FD_MRI) reached a peak in immune-excluded tumors (*ANOVA P* < 0.001; *post-hoc* Tukey test *P* < 0.001 vs. immune-inflamed and desert types) (**Figure 2A**). Metabolic fractal dimension (FD_PET) exhibited the highest readings in hypoxic immune-desert lesions (*ANOVA P* < 0.001; *P* < 0.01 vs. other types).

**Figure 2:**
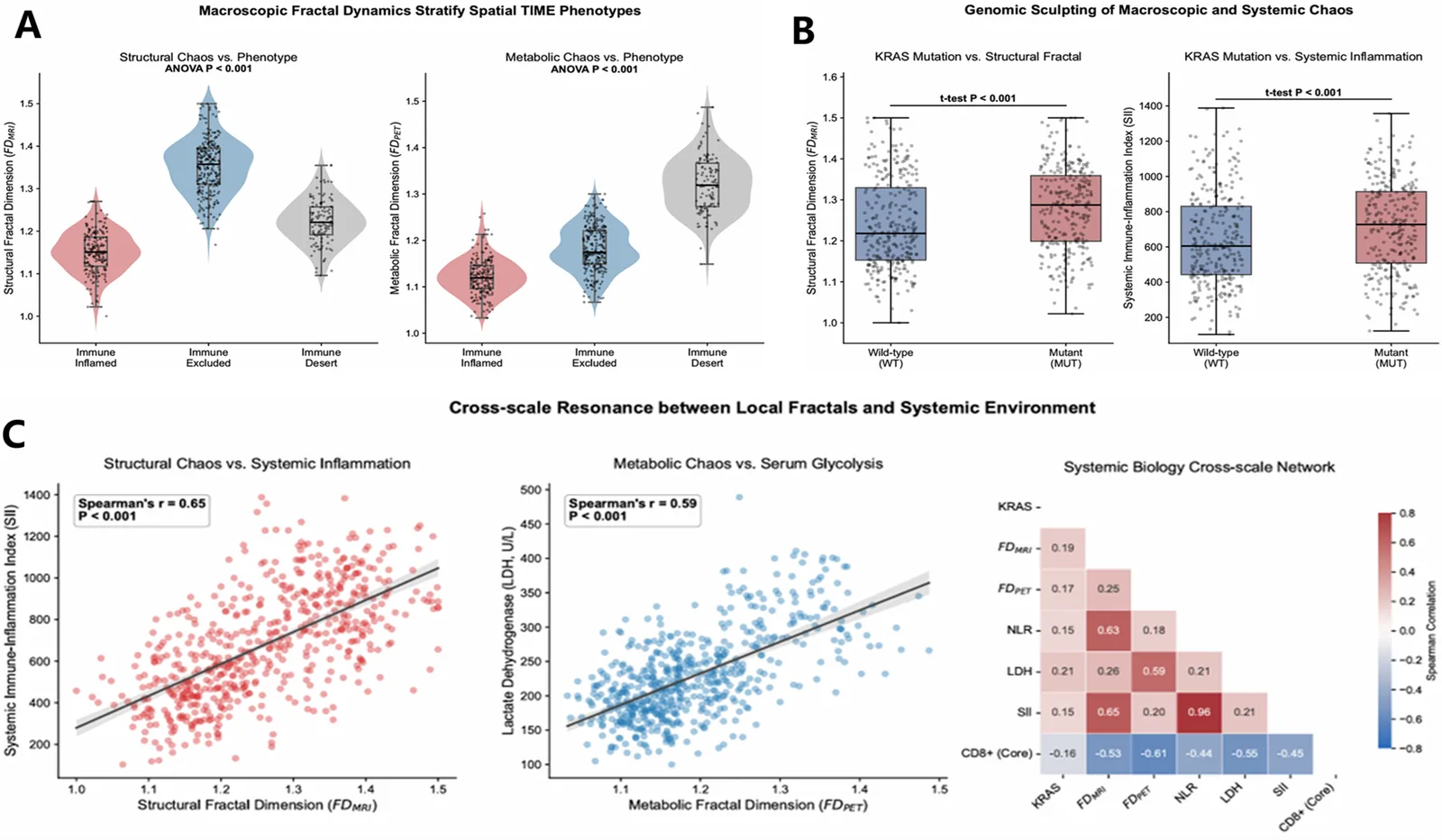
Stratification of spatial TIME phenotypes by macroscopic fractal dynamics and their associations with genomic and systemic profiles. **(A)** Violin plots demonstrating the stratification of spatial tumor immune microenvironment (TIME) phenotypes (immune-inflamed, immune-excluded, and immune-desert) based on the structural fractal dimension (FD_MRI) and metabolic fractal dimension (FD_PET) (*ANOVA P* < 0.001). The immune-excluded phenotype exhibits the highest FD_MRI, whereas the immune-desert phenotype displays the highest FD_PET. **(B)** Box plots illustrating the impact of genomic alterations on macroscopic and systemic states. Tumors harboring *KRAS* mutations (MUT) show significantly higher macroscopic structural complexity (FD_MRI) and systemic inflammatory burden (SII) compared to wild-type (WT) tumors (*t*-test *P* < 0.001). **(C)** Cross-scale correlation analysis. Scatter plots indicate significant positive correlations between FD_MRI and SII (Spearman *r* = 0.65, *P* < 0.001), and between FD_PET and serum lactate dehydrogenase (LDH) (Spearman *r* = 0.59, *P* < 0.001). The correlation heatmap summarizes the cross-scale network, mapping interactions among genomic drivers (*KRAS*), macroscopic fractals, systemic indicators (NLR, LDH, SII), and microscopic core CD8^+^ T cell infiltration. *P < 0.05, **P < 0.01, ***P < 0.001.

Systems biology analysis indicated a correlation between the local tumor microenvironment and the systemic immune network (**Figure 2B**). After correcting for tumor volume and background liver fibrosis, local structural physical state (FD_MRI) remained positively correlated with the peripheral blood systemic immune-inflammation index (SII) (Spearman’s *r* = 0.65, *P* < 0.001). Local energy metabolic disorder (FD_PET) was associated with an increase in peripheral serum lactate dehydrogenase (LDH) (Spearman’s *r* = 0.59, *P* < 0.001). Macroscopic imaging fractals and peripheral blood systemic exhaustion indices were negatively correlated with the absolute infiltration density of CD8^+^ T cells in the core region (FD_PET vs. CD8^+^: *r* = -0.61). The oncogenic driver stratified boxplot (**Figure 2C**) showed that *KRAS* mutant patients had higher local structural fractal (FD_MRI) and peripheral SII burden than wild-type patients (*t*-test *P* < 0.001).

Interaction term analysis indicated a synergistic inhibitory effect: Patients presenting with local structural characteristics (FD_MRI in the upper quartile) and systemic immune exhaustion (SII in the upper quartile) showed decreased infiltration density of CD8^+^ T cells at the tumor invasive margin and core region, supporting the concept of an immune sink mechanism affecting T cell infiltration.

### 3.3 SHAP decodes algorithmic mapping thresholds for spatial immune polarization

To address the interpretability limitation of machine learning classifiers, the SHAP analysis framework was used. The feature importance matrix (**Figure 3A**) showed that structural fractal dimension (FD_MRI, mean absolute SHAP value 1.892) and metabolic fractal dimension (FD_PET, 1.230) were the top two contributors to the classification model’s output, surpassing serological LDH (1.008) and SII (0.934). The SHAP bee swarm plot (**Figure 3B**) showed that nonlinear increases in structural and metabolic fractals pushed tumor probability towards cold tumors (immune-excluded/desert), while MSI-H status provided a positive inflammatory classification.

**Figure 3:**
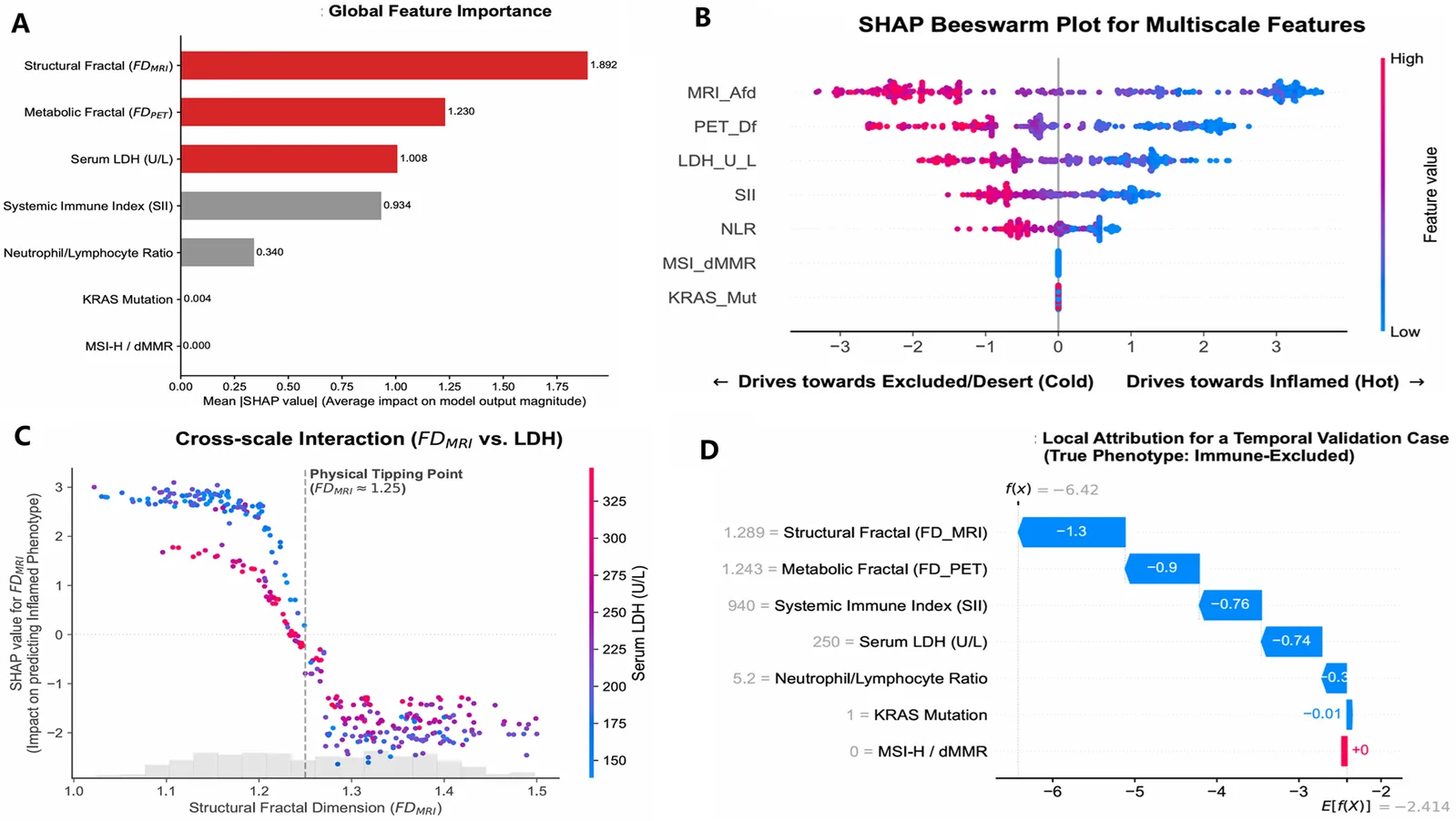
SHAP-based interpretation of the multi-scale machine learning architecture for predicting spatial TIME phenotypes. **(A)** Global feature importance ranked by mean absolute SHAP values. Macroscopic fractal dynamics (FD_MRI and FD_PET) and serum LDH emerge as the top three predictors in the joint model. **(B)** SHAP beeswarm plot illustrating the distribution of multi-scale features and their directional impact on model output. High values (magenta) of FD_MRI, FD_PET, LDH, and SII drive predictions toward the immune-excluded/desert (“cold tumor”) phenotype, while low values (blue) favor the immune-inflamed (“hot tumor”) phenotype. **(C)** SHAP dependence plot revealing the cross-scale interaction between FD_MRI and serum LDH. A non-linear physical threshold is observed near FD_MRI ≈ 1.25, beyond which the probability of predicting an inflamed phenotype decreases sharply; this suppressive effect is amplified by high LDH levels. **(D)** Local attribution waterfall plot deconstructing the prediction for a representative patient from the temporal validation cohort. Specific feature values (e.g., FD_MRI = 1.289, FD_PET = 1.243) cumulatively shift the baseline expected output E[f(x)] toward a definitive prediction f(x) of the immune-excluded phenotype, aligning with the pathological gold standard.

The SHAP interaction dependence plot (**Figure 3C**) identified an algorithmic mapping threshold: When FD_MRI exceeded ~1.25, the SHAP efficacy driving the tumor towards an immune-excluded state decreased (SHAP value < 0). When the lesion crossed this threshold accompanied by high peripheral serum LDH, the combined effect restricted T cells outside the core region. Independent SHAP projection analyses on the temporal validation cohort (2025) and external multi-center validation cohort showed this ~1.25 threshold exhibited cross-temporal and cross-spatial conservation.

As shown in the local attribution waterfall plot (**Figure 3D**) for an immune-excluded patient from the temporal validation cohort, the macroscopic physical barrier feature (FD_MRI = 1.289) contributed a weight of -1.3 to the model’s prediction log-odds. Combined with metabolic fractal (FD_PET = 1.243), SII (940), and LDH (250), the XGBoost algorithm captured the cold tumor phenotype.

### 3.4 Diagnostic advantages of the cross-scale radiomics-immune joint score

A multi-scale radiomics-immune joint nomogram score (IRJS) was constructed. The clinical and systemic immune model showed AUCs of 0.752, 0.728, and 0.714 across the three cohorts (**Table 2**). The joint model IRJS achieved an apparent AUC of 0.921 (95% CI: 0.88-0.96) for identifying immune-inflamed tumors in the training cohort, with an accuracy of 87.3%, sensitivity of 89.6%, specificity of 86.3%, and F1-Score of 0.885. To rigorously evaluate internal validity and mitigate the risk of overfitting, a 1, 000-iteration bootstrap resampling was performed; the optimism-corrected AUC remained highly robust at 0.903, confirming the model’s stability.

**Table 2:** Predictive performance of multiscale XGBoost models for identifying the immune-inflamed phenotype across cohorts.

Cohorts and models	AUC (95% CI)	Accuracy (%)	Sensitivity (%)	Specificity (%)	F1-Score	P-value (DeLong)*
Training cohort (n = 150)						
Clinical & Systemic Immune Model	0.752 (0.69–0.82)	68.7	64.6	70.6	0.658	< 0.001
Macroscopic Fractal Model	0.825 (0.76–0.88)	76.0	79.2	74.5	0.776	0.012
**Joint Multiscale Model (IRJS)**	**0.921 (0.88–0.96)**	**87.3**	**89.6**	**86.3**	**0.885**	**Reference**
Temporal validation cohort (n = 45)						
Clinical & Systemic Immune Model	0.728 (0.61–0.84)	66.7	62.5	69.0	0.645	0.008
Macroscopic Fractal Model	0.791 (0.68–0.89)	73.3	75.0	72.4	0.750	0.045
**Joint Multiscale Model (IRJS)**	**0.886 (0.80–0.97)**	**82.2**	**87.5**	**79.3**	**0.848**	**Reference**
External validation cohort (n = 75)						
Clinical & Systemic Immune Model	0.714 (0.62–0.81)	64.0	56.5	67.3	0.604	< 0.001
Macroscopic Fractal Model	0.773 (0.68–0.86)	72.0	73.9	71.1	0.731	0.038
**Joint Multiscale Model (IRJS)**	**0.862 (0.79–0.94)**	**80.0**	**82.6**	**78.8**	**0.817**	**Reference**

The Clinical & Systemic Immune Model integrates *KRAS/BRAF*/MSI status, NLR, SII, LDH, and CEA. The Macroscopic Fractal Model utilizes FD_MRI and FD_PET. The Joint Multiscale Model integrates all aforementioned features.

The *P*-value is calculated using the DeLong test, comparing the Area Under the Receiver Operating Characteristic Curve (AUC) of the Clinical/Systemic Immune Model or the Macroscopic Fractal Model against the Joint Multiscale Model (IRJS, set as the Reference).

IRJS, Immune-Radiomics Joint Score.

Bold values indicate the performance metrics of the Joint Multiscale Model (IRJS), the final integrative model developed in this study, which was set as the reference standard for the DeLong test comparisons.

In the temporal validation cohort (Center A, 2025) and the external validation cohort (Center B), the IRJS score maintained AUCs of 0.886 (95% CI: 0.80-0.97) and 0.862 (95% CI: 0.79-0.94), with classification accuracies of 82.2% and 80.0% (**Figure 4A**). To evaluate the necessity of this non-linear architecture, IRJS was benchmarked against an L1-penalized LASSO regression utilizing the identical feature set. In the temporal validation cohort, the LASSO model yielded an AUC of 0.825 (95% CI: 0.74-0.90). DeLong’s test confirmed that the XGBoost-based IRJS provided a statistically significant incremental improvement in discriminatory capacity (*P* = 0.034) (**Supplementary Table 4**), justifying its use in capturing complex biophysical threshold effects (e.g., the FD_MRI ≈ 1.25 tipping point). In the internal prospective temporal validation cohort (n=45) and the external validation cohort (n=75), the IRJS score demonstrated robust probability calibration (Brier Score 0.142 and 0.158, respectively; Hosmer-Lemeshow *P* = 0.68 and *P* = 0.55, respectively). DeLong test comparisons confirmed the diagnostic performance of the joint model IRJS was statistically superior to the clinical systemic immune model (*P* < 0.01) and the pure imaging fractal model (P < 0.05) across validation settings (**Table 2**).

**Figure 4:**
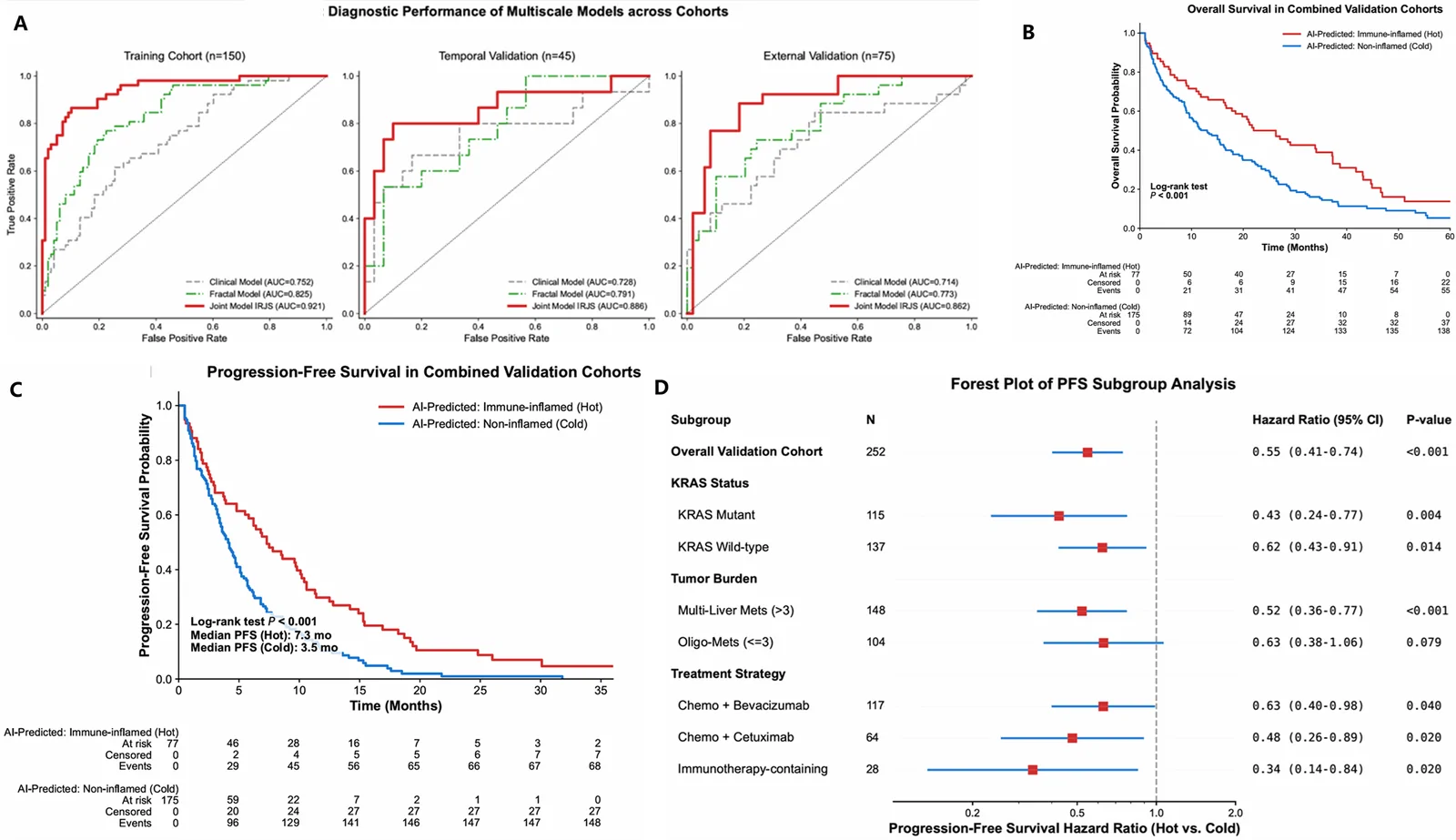
Diagnostic performance and prognostic validation of the Immune-Radiomics Joint Score (IRJS) across independent cohorts. **(A)** Receiver operating characteristic (ROC) curves evaluating the performance of the baseline clinical model, macroscopic fractal model, and joint model (IRJS) in identifying the immune-inflamed phenotype. IRJS consistently outperformed single-dimension models in the training (AUC = 0.921), temporal validation (AUC = 0.886), and external validation (AUC = 0.862) cohorts. **(B)** Kaplan-Meier overall survival (OS) analysis in the combined validation cohorts. Patients classified as AI-predicted immune-inflamed (“hot tumor”) exhibited significantly longer OS than non-inflamed (“cold tumor”) patients (Log-rank test *P* < 0.001). **(C)** Kaplan-Meier progression-free survival (PFS) analysis in the combined validation cohorts. The AI-predicted “hot tumor” phenotype was associated with a prolonged median PFS (7.3 months) compared to the “cold tumor” phenotype (3.5 months) (Log-rank test *P* < 0.001). **(D)** Forest plot of PFS subgroup analyses. The prognostic advantage of the AI-predicted “hot tumor” phenotype (hazard ratio < 1.0) remained consistent across clinical stratifications, including *KRAS* mutation status, liver tumor burden, and specific systemic treatments (e.g., chemotherapy plus bevacizumab, immunotherapy-containing regimens).

### 3.5 Prognostic survival stratification and clinical nomogram deployment

During survival follow-up of the combined validation cohort (n=252), the spatial immune stratification predicted by the IRJS score yielded significant survival outcome separation. Kaplan-Meier survival curves (**Figure 4B**) revealed that OS curves for AI-predicted immune-inflamed and non-inflamed patients exhibited separation (Log-rank test *P* < 0.001).

Univariate and multivariate Cox proportional hazards regression analyses (**Table 3**; **Figure 5A**) adjusted for age, primary tumor site, metastatic burden (>3 liver metastases), and *KRAS* mutation status. The non-inflamed phenotype predicted by IRJS was confirmed as an independent risk factor for post-treatment mortality (adjusted HR = 3.25, 95% CI: 2.15–4.85, *P* < 0.001). The AI-identified immune-inflamed phenotype was an independent survival-protective biomarker (HR = 0.31, 95% CI: 0.21–0.47, *P* < 0.001).

**Table 3:** Univariate and multivariate Cox proportional hazards regression analysis for overall survival (OS) in the combined validation cohorts.

Variables	Univariate analysis HR (95% CI)	P-value	Multivariate analysis adjusted HR (95% CI)	P-value
Clinical and pathological factors				
Age (> 65 *vs.* ≤ 65 years)	1.12 (0.85–1.48)	0.425	1.15 (0.82–1.55)	0.420
Sex (Male *vs.* Female)	0.95 (0.72–1.25)	0.708	–	–
Primary tumor location (Right *vs.* Left)	1.45 (1.10–1.92)	0.009	1.42 (1.05–1.95)	0.021
Node Status (N+ *vs.* N0)	1.74 (1.25–2.42)	0.001	1.65 (1.18–2.30)	0.003
Hepatic Burden (> 3 *vs.* ≤ 3)	1.68 (1.25–2.26)	< 0.001	1.35 (0.95–1.88)	0.095
Genomic and systemic indices				
*KRAS* mutation (Mutant vs. Wild-type)	1.75 (1.32–2.34)	< 0.001	1.58 (1.12–2.15)	0.008
Baseline CEA (> 50 *vs.* ≤ 50 ng/mL)	1.38 (1.05–1.82)	0.021	1.15 (0.85–1.55)	0.362
Systemic Immune-Inflammation Index (High *vs.* Low) *	1.62 (1.20–2.18)	0.002	1.38 (0.95–1.98)	0.085
AI-derived spatial immune phenotype				
Model-predicted Phenotype (Non-inflamed *vs.* Immune-inflamed) **	2.86 (1.92–4.17)	< 0.001	3.25 (2.15–4.85)	< 0.001

HR, Hazard Ratio; CI, Confidence Interval; CEA, Carcinoembryonic Antigen.

Variables with *P* < 0.10 in the univariate analysis, alongside predetermined clinically relevant covariates (e.g., Age), were rigorously included in the multivariate Cox proportional hazards regression model to eliminate confounding collinearity.

(*) The cutoff for SII (Systemic Immune-Inflammation Index) was determined using the median value of the training cohort.

(**) The “Model-predicted Phenotype” is the binary output (Immune-inflamed vs. Non-inflamed/Excluded/Desert) generated by the Joint Multiscale XGBoost Model (IRJS).

**Figure 5:**
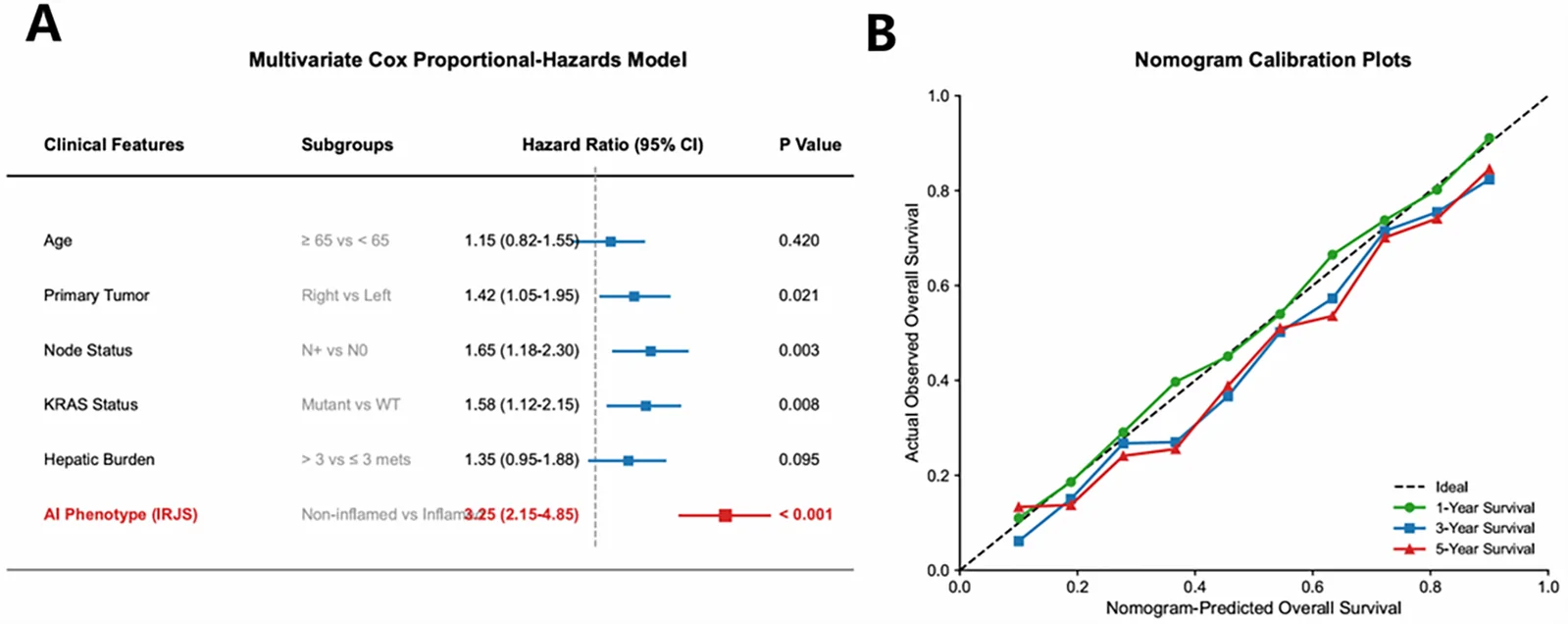
Independent prognostic value and survival calibration of the Immune-Radiomics Joint Score (IRJS). **(A)** Forest plot of the multivariate Cox proportional hazards regression analysis. After adjusting for clinicopathological covariates (age, primary tumor location, lymph node status, *KRAS* mutation status, and liver metastatic burden), the IRJS-predicted non-inflamed phenotype was identified as the significant independent risk factor for overall survival (OS) (adjusted HR = 3.25, 95% CI: 2.15–4.85, *P* < 0.001). **(B)** Calibration curves for predicting 1-year (green), 3-year (blue), and 5-year (red) OS probabilities using the integrated IRJS nomogram. Nomogram-predicted survival probabilities (X-axis) showed high concordance with actual observed survival proportions (Y-axis) across all follow-up time points, closely adhering to the 45-degree reference line.

The clinical companion nomogram based on this joint score demonstrated concordance in predicting 1-, 3-, and 5-year survival rates (**Figure 5B**).

### 3.6 Clinical translational value: guiding treatment response and longitudinal monitoring

An efficacy stratification analysis in the treatment cohort (n=252) assessed PFS between survival curves (median PFS: 7.3 months vs. 3.5 months; PFS HR = 0.55, 95% CI: 0.41–0.74, *P* < 0.001) (**Table 4**; **Figure 4C**).

**Table 4:** Association of AI-predicted spatial immune phenotypes with progression-free survival (PFS) across systemic treatment regimens.

Treatment regimens and subgroups	N	Median PFS (months) inflamed *vs.* non-inflamed	PFS hazard ratio (95% CI)	PFS P-value
**Overall Validation Cohorts**	**252**	**7.3 vs. 3.5**	**0.55 (0.41–0.74)**	**< 0.001**
Stratified by Treatment Strategy*				
Chemotherapy + Bevacizumab	117	—	0.63 (0.40–0.98)	0.040
Chemotherapy + Cetuximab **	64	—	0.48 (0.26–0.89)	0.020
Immunotherapy-containing ***	28	—	0.34 (0.14–0.84)	0.020

*The overall validation cohorts refer to the combined training, temporal validation, and external validation cohorts with complete survival and follow-up data (Total N = 252).

Only patients with *KRAS*/*NRAS*/*BRAF* wild-type status were eligible for Cetuximab.

***Immunotherapy-containing regimens typically included PD-1/PD-L1 inhibitors combined with targeted therapy or chemotherapy.

Bold values indicate the PFS results across the overall combined validation cohorts (total N = 252), prior to stratification by treatment strategy.

The PFS subgroup analysis (**Table 4**; **Figure 4D**) demonstrated IRJS performance across treatment strategies:

- Chemotherapy + Bevacizumab (n=117): AI-predicted inflamed patients showed prolonged median PFS (HR = 0.63, 95% CI: 0.40–0.98, *P* = 0.040).
- Chemotherapy + Cetuximab (n=64): The AI-identified inflamed phenotype showed an association with PFS (HR = 0.48, 95% CI: 0.26–0.89, *P* = 0.020).
- It must be emphasized that due to the limited sample size (n=28), the predictive value of IRJS in the immunotherapy subgroup is strictly exploratory. Immune checkpoint blockade (ICB)-containing regimen (n=28): In patients receiving anti-PD-1/PD-L1 combination therapy, the risk of disease progression was reduced in the inflamed group (PFS HR = 0.34, 95% CI: 0.14–0.84, *P* = 0.020), while the non-inflamed group exhibited primary immune resistance.

In longitudinal dynamics tracking (**Figure 6A**), patients achieving clinical response (lesion shrinkage >30% and crossing the PR/CR threshold) were identified as immune-inflamed by AI. The swimmer plot of treatment trajectories (**Figure 6B**) showed AI-predicted inflamed patients had durable responses, while non-inflamed tumors crossed the progression of disease (PD) threshold early.

**Figure 6:**
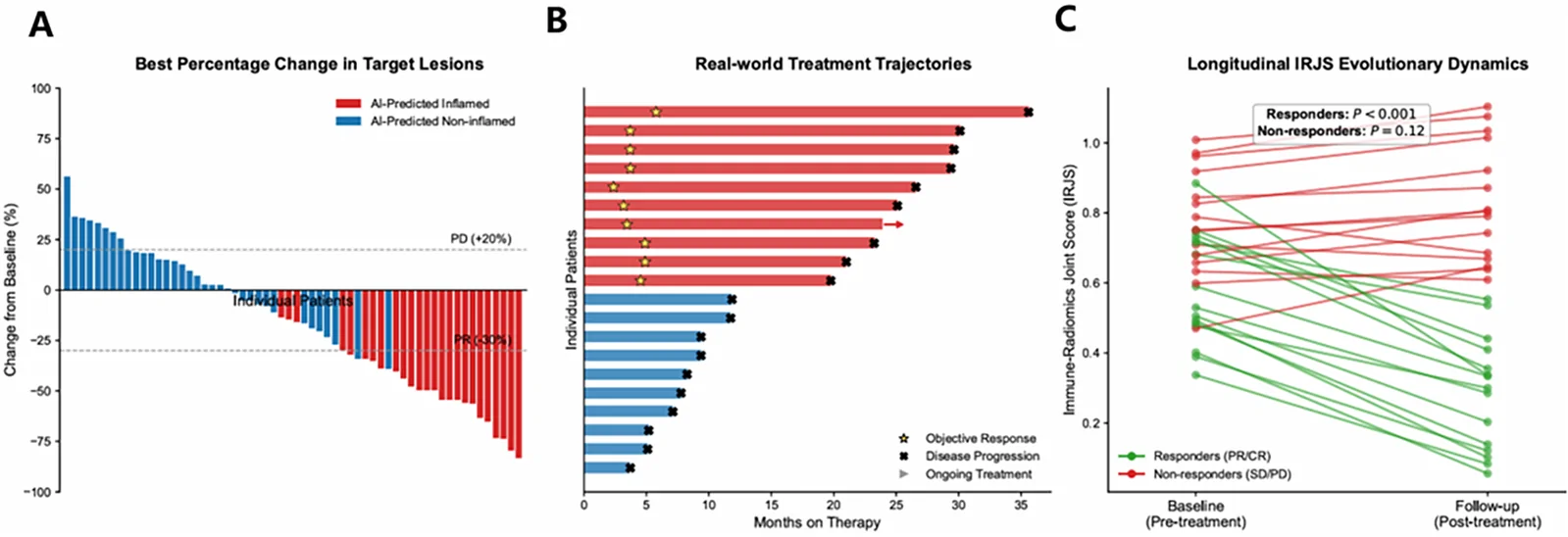
Treatment response prediction and longitudinal dynamics of the Immune-Radiomics Joint Score (IRJS). **(A)** Waterfall plot of the best percentage change in target lesion size relative to baseline in the validation cohorts. The AI-predicted immune-inflamed phenotype (red bars) showed a significantly higher objective response rate compared to the non-inflamed phenotype (blue bars). Dashed lines indicate RECIST 1.1 thresholds for partial response (PR, -30%) and progressive disease (PD, + 20%). **(B)** Swimmer plot of individual real-world treatment trajectories. The AI-predicted immune-inflamed group (red bars) demonstrated more durable clinical benefits and frequent objective responses (yellow stars) prior to disease progression (black crosses) than the non-inflamed group (blue bars). **(C)** Longitudinal dynamics of the IRJS during systemic therapy. From baseline to post-treatment follow-up, clinical responders (PR/CR, green line) exhibited a significant quantitative decline in IRJS (P < 0.001), reflecting the therapeutic disruption of macroscopic physical barriers and metabolic chaos. Conversely, clinical non-responders (SD/PD, red line) maintained high biological stress without significant fractal drift (*P* = 0.12).

Dynamic tracking of lesions (**Figure 6C**) showed that for clinical responders (PR/CR), the systemic IRJS score and core fractal dimensions exhibited a quantitative decrease after systemic intervention. In partial correlation analyses, after controlling for target lesion volume shrinkage rate (ΔVolume), the decrease in ΔFD maintained statistical significance (*P* < 0.001). This analysis demonstrates that longitudinal fractal drift reflects the structural alteration of the physical mechanical stress wall by anti-angiogenic/chemotherapy regimens. For non-responders with acquired resistance, their multi-scale joint score showed high levels or rebound during the resistance window (*P* = 0.12).

### 3.7 Clinical utility, reclassification improvement, and net benefit assessment

To evaluate the application of the IRJS in multidisciplinary team (MDT) scenarios, continuous net reclassification improvement (NRI), integrated discrimination improvement (IDI), and decision curve analysis (DCA) were calculated.

Compared to the baseline clinical model, the IRJS integrating fractal dynamics achieved net reclassification improvement (NRI = 0.384, *P* < 0.001) and integrated discrimination improvement (IDI = 0.256, *P* < 0.001). This indicates that introducing macroscopic spatial physical fractal dynamics features (FD_MRI and FD_PET) can correct misclassifications of non-inflamed patients that would occur based solely on peripheral serological systemic inflammation indices (SII, NLR) or single driver gene status.

Clinical decision curves (DCA) quantified the net benefit of this tool. The joint nomogram score demonstrated utility across a threshold probability range of 10% to 90% compared to “treat-all” and “treat-none” strategies and the clinical systemic immune model. At the 50% threshold, the joint decision tool elevated the clinical net benefit rate compared to the baseline model, assisting MDT expert teams in biopsy decisions and intercepting primary immunotherapy resistance.

## 4 Discussion

The spatial tumor immune microenvironment (TIME) within solid tumors is a key factor determining clinical responses to immunotherapy and targeted therapy. Traditional biopsy relying on a single puncture is susceptible to spatial sampling bias, and conventional radiomics is often limited by a lack of interpretability (21). We evaluated a multi-scale immune-radiomics joint score (IRJS) across spatiotemporally independent cohorts to decode the spatial immune landscape of CRLM. The study indicated that macroscopic imaging fractal dimensions (FD_MRI and FD_PET) can map microscopic mechanical physical barriers and metabolic states. By integrating peripheral blood immune indices and genetic status, IRJS demonstrated phenotype discriminative ability and prognostic relevance.

### 4.1 Biomechanical mapping of macroscopic physical structures and spatial immune exclusion

A clinical observation of this study is that a high structural fractal dimension (FD_MRI) is associated with the microscopic “immune-excluded” state. FD_MRI is proposed to serve as a macroscopic surrogate for the physical stress wall associated with the deposition of α-SMA^+^ pro-fibrotic stroma and a malformed CD31^+^ microvascular network. While α-SMA identifies the presence of activated cancer-associated fibroblasts (CAFs), the actual mechanical exclusion of T cells is associated with by the subsequent remodeling of the extracellular matrix (ECM), including the dense deposition of Collagen I/III and Fibronectin. FD_MRI exhibited a positive correlation with α-SMA-marked pro-fibrotic stromal density (*r* = 0.684, P < 0.001) and was consistent with the spatial rejection of CD8^+^ effector T cells at the invasive margin. This macroscopic observation aligns with the hypothesis that proliferating cancer-associated fibroblasts (CAFs) and disorganized vascular networks may construct a rigid physical barrier, thereby restricting the passive diffusion of CD8^+^ T cells (23).

Using the SHAP algorithm, we evaluated an algorithmic mapping threshold. When FD_MRI crossed the threshold of ~1.25, the SHAP value driving the tumor towards an immune-excluded state decreased below 0, suggesting a limit affecting T cell transvascular migration. Macroscopic topological fractals may serve as a surrogate marker for interstitial fluid pressure, providing a quantification paradigm for tumor stromal stiffness. It is important to note that the spatial complexity of the CRLM microenvironment is not solely defined by adaptive immune cells like CD8^+^ T cells; the remodeling of the TIME also involves the dynamic trafficking of innate immune populations, such as the chemotaxis of N1 neutrophils, which play a distinct role in limiting metastatic progression (22). This ~1.25 threshold is a mathematical weight allocated by SHAP based on the XGBoost loss function and serves as a hypothesis for future experiments in tumor mechanobiology.

### 4.2 Metabolic fractal features recapitulate the local “metabolic desert” and systemic immune sink

Energy metabolic competition and hypoxia shape the “immune-desert” phenotype (24). We adopted an extraction strategy processing PET and MRI masks in independent three-dimensional spaces to support the geometric fidelity of fractal dimension calculation.

Tumor core regions exhibiting high FD_PET fractal readings were spatially coincident with HIF-1α enrichment and CD8^+^ T cell deficiency. The metabolic fractal state captured by macroscopic imaging maps the anaerobic glycolysis and hypoxic stress dominated by malignant clones and suppressive stroma. This aligns with the consensus that anaerobic glycolysis (*Warburg* effect) leads to local glucose scarcity and lactic acid accumulation (25). This state, associated with Wnt/β-catenin signaling, relates to the local hypoxia driven by the Warburg effect (26). This metabolic environment impairs effector T cell function through substrate deprivation and disrupts the PI3K/Akt/mTOR metabolic signaling axis. While our current spatial pathology data primarily demonstrates the physical depletion of CD8^+^ T cells in the tumor core, this macroscopic metabolic chaos (high FD_PET) strongly aligns with theoretical models suggesting the upregulation of co-inhibitory receptors (e.g., PD-1, TIM-3) and the polarization of infiltrating T cells towards an exhausted phenotype (27, 28).

Local structural and metabolic fractal features (high FD_MRI and high FD_PET) were accompanied by an increase in peripheral blood systemic immune-inflammation indices (SII, NLR) and a decrease in circulating CD8^+^ T cells. The interaction between high systemic inflammatory stress (e.g., elevated SII/LDH) and high FD_PET metabolic heterogeneity suggests a potential “immune sink” dynamic. We hypothesize that systemic metabolic reprogramming may trap T cells in the peripheral circulation, accelerating exhaustion before they can penetrate the local tumor barrier (29). Furthermore, our model demonstrates that *KRAS* mutations are significantly associated with specific macro-radiomic phenotypes, supporting existing biological theories that oncogenic *KRAS* signaling may promote an immunosuppressive stroma. However, it is crucial to emphasize that our findings are derived from observational macro-imaging data; the causal molecular mechanisms driving these associations remain to be definitively established. The IRJS model integrates local imaging fractals with peripheral blood host immune exhaustion indicators to assess this process.

### 4.3 Clinical decision deployment of the multi-scale model

The multi-scale joint score (IRJS) demonstrated discriminative utility compared to traditional single-dimension clinical models (as shown in **Figure 7**). Across validation cohorts, the AUC of IRJS for identifying immune-inflamed tumors remained between 0.862–0.921, surpassing the clinical-genomic model (AUC 0.714–0.752). After adjusting for variables including age, primary tumor site, metastatic burden, and *KRAS* mutation status, the non-inflamed phenotype predicted by IRJS was an independent risk factor for post-treatment mortality in CRLM patients (adjusted HR 3.25, 95% CI: 2.15–4.85, *P* < 0.001). The AI-identified immune-inflamed phenotype was an independent protective biomarker (HR = 0.31, 95% CI: 0.21–0.47, *P* < 0.001).

**Figure 7:**
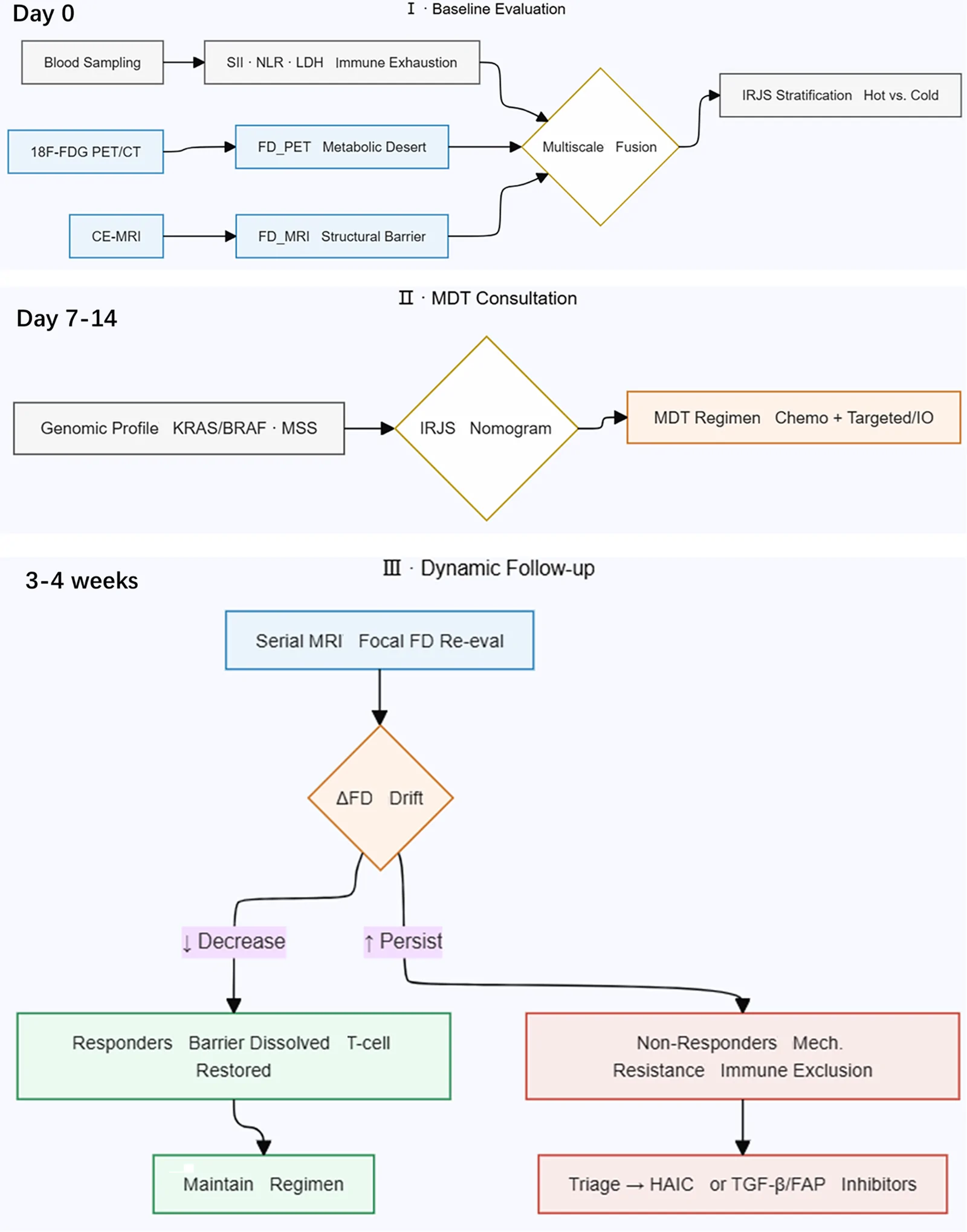
Integration of the Immune-Radiomics Joint Score (IRJS) into the multidisciplinary team (MDT) clinical decision workflow. *Phase I: Baseline Assessment (Day 0).* Initial non-invasive immune stratification is performed using peripheral blood sampling for systemic immune exhaustion indicators (SII, NLR, LDH) and macroscopic fractal dynamics extracted from ^18^F-FDG PET/CT (FD_PET, mapping the metabolic desert) and CE-MRI (FD_MRI, mapping the structural barrier). Multi-scale fusion of these parameters allows for rapid preliminary classification of the phenotype as “hot” (immune-inflamed) or “cold” (immune-excluded/desert) prior to biopsy results. *Phase II: MDT Consultation (Days 7–14).* Following genomic analysis (e.g., *KRAS*/*BRAF* mutations, MSS status), variables are incorporated to generate the precise IRJS nomogram, guiding the MDT in formulating individualized systemic regimens (e.g., chemotherapy plus targeted therapy or immuno-oncology [IO] drugs). *Phase III: Dynamic Follow-up (Weeks 3–4).* Early fractal drift (ΔFD) is evaluated via continuous MRI monitoring. A quantitative decrease in FD values (↓) indicates the disruption of physical barriers and restoration of T cell infiltration in early responders, supporting treatment continuation. Persistently high or rebounding FD values (↑) suggest mechanical resistance and sustained immune exclusion in non-responders, facilitating the timely triage of patients to high-intensity regional interventional therapies (e.g., HAIC) or clinical trials (e.g., TGF-*β* or FAP inhibitors) to intercept primary resistance.

We outline an application of IRJS within the multidisciplinary team (MDT) pathway:

- Initial Diagnosis Day (Day 0): Clinicians can assess the physical barrier and metabolic profile of the target lesion for an initial immune qualitative assessment (Base-IRJS) using CE-MRI and PET/CT fractal readings and peripheral blood inflammation indices (SII, LDH).
- Multidisciplinary Consultation Day (Day 14): After molecular NGS reports, the MDT incorporates the *KRAS* mutant variable to update the IRJS score, integrating radiological, pathological, and oncological data into the clinical decision cycle.

For multi-center application, the numerical value derived from the training set using the Youden index was applied across different centers and devices. While our preprocessing pipeline (isotropic resampling) successfully maintained predictive robustness (AUC 0.862) across distinct MRI vendors (Siemens vs. GE) without explicit statistical alignment, application remains susceptible to major magnetic field strength differences (1.5T vs. 3.0T) and extreme reconstruction algorithms (30). The future clinical direction is not to forcefully seek an absolute numerical boundary, but to integrate advanced batch-effect correction algorithms (e.g., ComBat harmonization) and establish a fractal normative database based on multi-center samples. This would output the ‘spatial immune exclusion risk percentile’ of the patient’s combined fractal indicators within a population norm. Subsequent iterations may encapsulate this cross-scale fractal algorithm into an imaging navigation module, interfacing with medical image automatic segmentation models (e.g., SAM) for delineation of lesion boundaries (31).

### 4.4 Longitudinal microenvironment remodeling and efficacy follow-up

IRJS was evaluated as a dynamic assessment of spatial microenvironment remodeling. In the early post-intervention period, responders achieving objective response (ORR) exhibited a decrease in systemic IRJS score and core fractal dimensions (paired *t*-test *P* < 0.001). In partial correlation analyses, controlling for target lesion volume shrinkage rate (ΔVolume), the decrease in ΔFD maintained statistical significance (*P* < 0.001). This indicates that longitudinal fractal drift reflects the deconstruction of the physical mechanical barrier by anti-angiogenic agents or systemic chemotherapy, reflecting the normalization of microvasculature and matrix remodeling to support T cell infiltration (32). The IRJS score may serve as an *in vivo* follow-up tool for tracking spatial immune evolution.

The observed week 3 fractal drift occurs earlier than the timeframe recommended by international guidelines for routine imaging efficacy evaluation (6-8 weeks). Requiring patients to undergo additional CE-MRI or PET/CT examinations early in treatment has health economics implications (33). We suggest developing tailored sequences, such as rapid, contrast-agent-free DWI or MRI volumetric scanning protocols, to reduce the cost of dynamic monitoring. For patients whose IRJS score remains high or rebounds early after systemic intervention (weeks 3-4), the MDT can triage patients to regional treatments (such as HAIC) or clinical trials targeting fibroblast activation protein (FAP) and TGF-*β* inhibitors.

### 4.5 Spatial evolution of physical matrix constraint and immune evasion

This study addresses a mechanistic question: Does the matrix barrier induced by oncogenic mutations mechanically repel T cells, or does the lack of T cells permit the proliferation of physical structures? We propose a bidirectional dynamic feedback loop present throughout tumor evolution (as shown in **Figure 8**):

**Figure 8:**
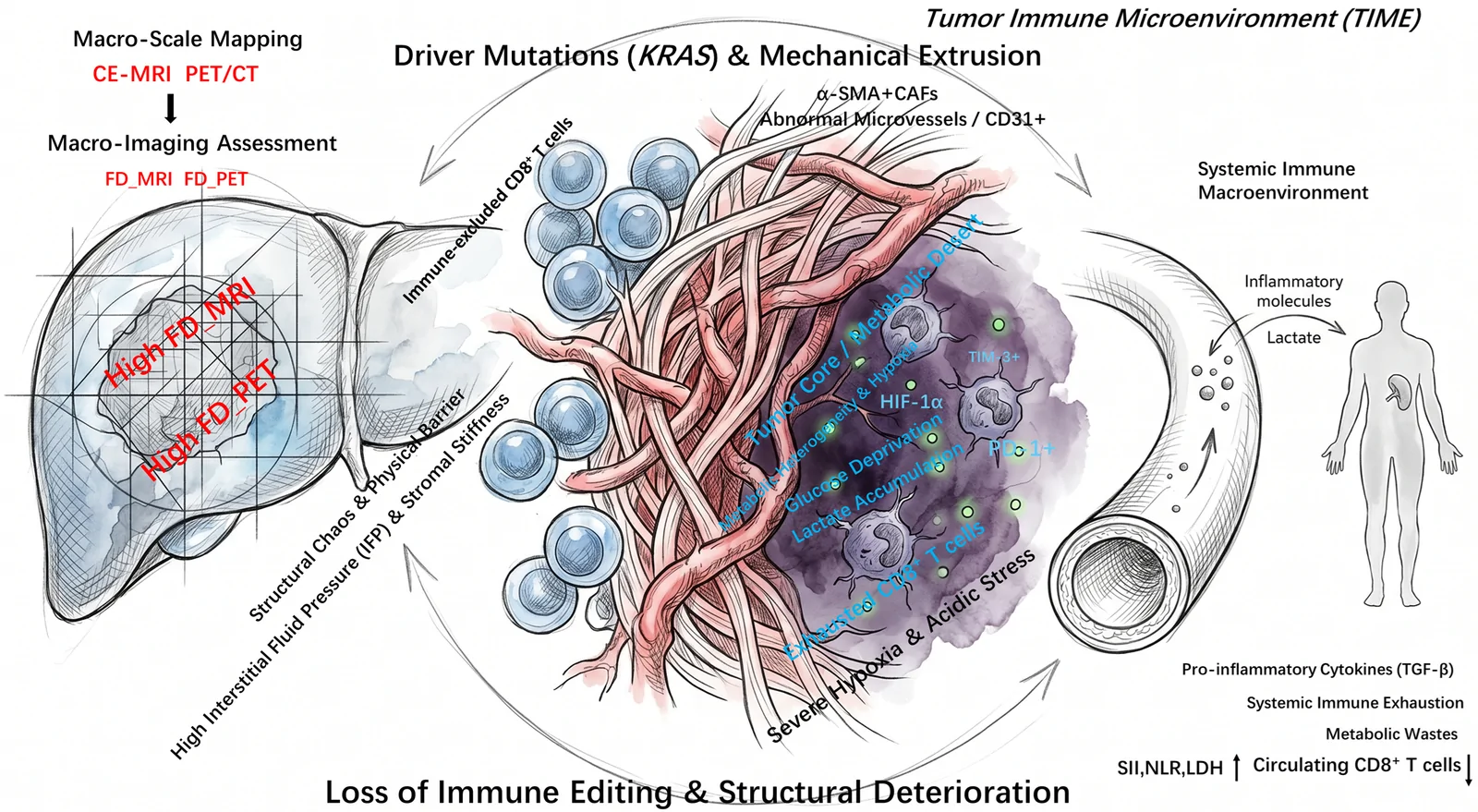
Working model of the cross-scale physical-immune coupling mechanism and bidirectional feedback loop. *Macroscopic mapping:* The left panel illustrates non-invasive imaging assessments of liver lesions using CE-MRI (portal venous phase) and ^18^F-FDG PET/CT. High FD_MRI maps extreme structural chaos and physical barrier formation, while high FD_PET reflects severe intratumoral metabolic heterogeneity and hypoxia. *Microscopic microenvironment sculpting:* The central schematic decodes the tumor immune microenvironment (TIME). The outer layer forms a physical wall constructed by α-SMA^+^ cancer-associated fibroblasts (CAFs) and malformed microvessels (CD31^+^), generating high interstitial fluid pressure (IFP) and stromal stiffness that trap effector cells at the invasive margin (immune-excluded CD8^+^ T cells). The tumor core is characterized by hypoxia and acidic stress; the accumulation of HIF-1α and lactate, alongside glucose deprivation, induces dysfunction in sparsely infiltrating cells, leading to exhausted CD8^+^ T cells expressing high levels of PD-1^+^ and TIM-3^+^. *Systemic immune trap:* The right panel depicts the systemic immune macroenvironment. Local stress releases pro-inflammatory cytokines (e.g., TGF-*β*) and metabolic wastes into the peripheral circulation, triggering systemic immune exhaustion characterized by elevated inflammatory indices (SII, NLR, LDH) and a decline in circulating CD8^+^ T cells. *Bidirectional feedback loop:* Initial driver mutations (e.g., *KRAS*) initiate stromal activation and mechanical exclusion. The resulting physical exclusion of T cells associates with a loss of immune editing capacity, which subsequently permits further structural deterioration and fractal chaos.

#### 4.5.1 Initiation causal stage

We hypothesize that in early metastasis, secretion of pro-fibrotic cytokines mediated by driver genes (e.g., *KRAS* mutations) induces a malformed microvascular network and desmoplastic reaction (high FD_MRI), constructing a spatial mechanical wall that limits immune cells (34).

#### 4.5.2 Negative feedback acceleration stage

The functional absence of immune editing associates with the microenvironment to lose mechanisms to clear stromal cells and suppress glycolysis (35). Left unchecked, stromal stiffening and anaerobic glycolysis (high FD_PET) exacerbate the nonlinear deterioration of the macroscopic fractal structure.

The IRJS score captures the cross-section of this bidirectional feedback loop at clinical consultation. Future tracking of liver metastases relying on spatial transcriptomics may further evaluate this loop of mechano-immune co-evolution.

### 4.6 Limitations and future perspectives

Limitations of this study include: First, while a 2025 prospective validation cohort was included, the study is fundamentally observational and largely retrospective. This design precludes the establishment of definitive causal biological mechanisms; hypotheses generated in this study—such as the ‘immune sink mechanism’ or the mechanical exclusion of T cells—rely strictly on macroscopic imaging correlations and require rigorous validation through future multi-omic and spatial transcriptomic mechanistic studies. Furthermore, our strict inclusion criteria—specifically the requirement for concurrent baseline CE-MRI and ^18^F-FDG PET/CT—inherently introduce selection bias, as this may exclude patients with rapid disease progression or those lacking access to comprehensive dual-modality imaging. Consequently, prospective randomized controlled trials (RCTs) are needed to definitively establish the role of IRJS in clinical decision-making. Second, the multi-scale model strictly relies on dual-modality input of CE-MRI and ^18^F-FDG PET/CT, which may present accessibility and cost constraints. Future work will aim to develop single-modality models (e.g., using CE-MRI and blood SII/LDH). Third, the sample size of the immune checkpoint blockade (ICB) subgroup is small (n=28), rendering the findings on ICB resistance exploratory. Although the efficacy stratification achieved statistical significance, the current evidence primarily supports the hypothesis that IRJS as a general prognostic indicator rather than a treatment-specific predictive biomarker. Validating its predictive value for ICB efficacy will require large-scale, prospective cohorts. Fourth, *KRAS* mutation was evaluated as a binary variable. Different *KRAS* mutation subtypes exhibit varied effects on the microenvironment (e.g., G12D tends to enrich MDSCs) (36). We will explore subtype-specific integrated models in future cohorts. Fifth, while this study supports the hypothesis that a robust statistical correlation between macroscopic fractal dimensions and microscopic digital pathology phenotypes (such as α-SMA and HIF-1α enrichment), it inherently lacks definitive mechanistic validation. Correlation does not equate to direct biological causation. The current retrospective design precludes the use of advanced biophysical and molecular interrogations necessary to prove that fractal features directly capture extracellular matrix stiffness or functional immune exhaustion. Future prospective investigations must employ a multi-dimensional mechanistic approach: (1) utilizing specialized extracellular matrix assays, such as Masson’s trichrome staining, Fibroblast Activation Protein (FAP) profiling, and Second-Harmonic Generation (SHG) microscopy, to quantitatively assess collagen I/III cross-linking architecture and ECM density; (2) incorporating high-dimensional multiplexed immunofluorescence to directly quantify functional exhaustion markers (e.g., PD-1, TIM-3, LAG-3, TOX) and effector cytokine production (e.g., IFN-γ, Granzyme B); (3) employing spatially resolved transcriptomics and single-cell RNA sequencing (scRNA-seq) to directly evaluate downstream oncogenic signaling cascades (e.g., the *KRAS*/TGF-*β* regulatory axis) and decode the precise metabolic signaling networks; and (4) performing *in vivo* needle manometry to measure absolute interstitial fluid pressure (IFP) and solid-phase mechanics (37). These rigorous validations are essential to bridge the translational gap between macroscopic multimodal imaging metrics and the intrinsic physical topology of the tumor microenvironment. Sixth, patients often receive systemic therapy (e.g., oxaliplatin) before liver metastasis biopsy, which can associate with background liver parenchymal changes and stromal stiffening, potentially introducing interference to structural fractal (FD_MRI) readings. Future models may introduce the fractal dimension of distal normal liver parenchyma to calculate a tumor-to-background liver fractal dimension ratio. Seventh, the survival analyses remain susceptible to residual confounding. While our multivariable Cox models adjusted for key clinical covariates—including categorical hepatic burden (>3 vs. ≤3 metastases) and *KRAS* status—we were constrained by the Events Per Variable (EPV) threshold from incorporating an exhaustive list of prognostic factors. Specific baseline systemic treatment regimens (e.g., various combinations of chemotherapy, targeted therapy, and immunotherapy) were not included as covariates in the multivariable OS analysis to prevent model overfitting. Critical variables such as exact volumetric total tumor burden (TTB), the resection status of the primary tumor, and the heterogeneity of both baseline and subsequent lines of systemic therapy were not fully adjusted for, which may confound the long-term survival outcomes. Future research utilizing larger registries is necessary to adjust for these comprehensive clinical dimensions and develop a volumetrically weighted fractal composite index based on total liver metastatic burden. Eighth, ^18^F-FDG PET/CT has limitations in spatial resolution, and partial volume effects may introduce noise into FD_PET readings. Moreover, the mathematical extraction of fractal dimensions is inherently sensitive to lesion-size effects; exceptionally small liver metastases (e.g., < 1 cm in diameter) encompass insufficient voxel counts, which can lead to topological instability and skewed fractal readouts. The structural barrier feature (FD_MRI) relies on CE-MRI for detailed characterization of liver lesions. Ninth, spatial sampling bias constitutes an inherent limitation. While macroscopic fractal features (3D-ROIs) were extracted from the complete tumor volume, the pathological reference standard for 24.1% of the cohort was derived from core needle biopsies (CNBs). An internal sub-analysis indicated an 87.5% phenotype concordance between CNB and paired surgical resections; nevertheless, phenotypic misclassification remains a risk, particularly if the biopsy trajectory fails to adequately capture the invasive margin. To minimize this bias, Whole Slide Imaging (WSI) was utilized for comprehensive two-dimensional digital evaluation, and a stringent inclusion criterion requiring a >1 mm tumor-liver interface was applied to all CNB samples. Despite these quality control measures, intratumoral heterogeneity dictates that the model was trained against a reference standard subject to spatial constraints. Future validation of spatial mapping accuracy utilizing large-scale surgical cohorts and 3D whole-mount step-sectioning is required. Tenth, while the current IRJS model utilizes non-specific structural (CE-MRI) and metabolic (^18^F-FDG PET/CT) imaging as macroscopic surrogates, the future trajectory of immune phenotype assessment will increasingly rely on antigen-specific molecular imaging. Recent advancements, such as the integration of Near-Infrared Fluorescence and Cerenkov Luminescence dual-modality *in vivo* imaging for specific PD-L1 tracking, highlight the immense clinical potential of direct immune biomarker visualization (38). Future iterations of our diagnostic framework should aim to integrate such targeted molecular imaging modalities to directly map immune checkpoint expression alongside macroscopic physical barriers, thereby providing a more definitive and multidimensional evaluation of the tumor immune microenvironment. Eleventh, the assessment of feature reproducibility in this study was limited to an initial Intraclass Correlation Coefficient (ICC) analysis on a 50-case subset from the training cohort. While a standardized preprocessing pipeline (isotropic resampling) was applied to normalize spatial geometry, the comprehensive impact of differing scanner hardware, reconstruction algorithms, and contrast protocols, and manual region-of-interest (ROI) delineation variability on fractal dimension stability remains to be fully elucidated. Before clinical translation can be achieved, a large-scale, independent reproducibility study across multiple vendor platforms is mandatory. Furthermore, transitioning from semi-automatic region-of-interest delineation to fully automated, deep-learning-based segmentation will be necessary to eliminate subjective manual ROI variability and standardize the extraction pipeline. Twelfth, although an external validation cohort was incorporated to evaluate cross-hardware robustness (Siemens vs. GE MRI platforms), both participating centers are affiliated with the same university system and serve a demographically and geographically homogeneous patient population. Consequently, the generalizability of the IRJS model across diverse racial and ethnic populations, varying institutional standard-of-care practices, and wider technical disparities (such as 1.5T vs. 3.0T magnetic field strengths or highly divergent PET/CT reconstruction algorithms) remains unverified. Future multinational, multi-institutional prospective registries are required. Such studies must incorporate advanced statistical harmonization techniques (e.g., ComBat) to calibrate fractal dimensional drift across global clinical settings and ensure the universal applicability of the model.

## 5 Conclusions

Macroscopic multimodal imaging fractal features (FD_MRI and FD_PET) can serve as non-invasive surrogate markers for internal mechanical physical barriers and metabolic hypoxic environments in CRLM. By combining local imaging fractals, driver gene status, and systemic immune-inflammation indices, this study evaluated an immune-radiomics joint score (IRJS). The IRJS assesses the cross-scale coupling mechanisms of tumor spatial immune polarization and the systemic immune sink, demonstrating phenotype discriminative ability and prognostic stratification. Its longitudinal fractal drift characteristic provides a metric for monitoring microenvironmental remodeling. The IRJS evaluation approach offers a potential framework for multidisciplinary teams (MDT) to explore biopsy decisions, combination therapy selection, and the assessment of primary immunotherapy and targeted therapy resistance. However, rigorous validation through prospective randomized controlled trials is required before this approach can be integrated into routine clinical decision-making.

## Data Availability

The raw data supporting the conclusions of this article will be made available by the authors, without undue reservation.
