# Supplementary material for: Mapping the physical topology of the tumor immune microenvironment: macroscopic image fractals as surrogate markers for immune exclusion phenotype and treatment resistance in colorectal cancer liver metastases
Source: Front Immunol. 2026 Sep 17;17:1889571. doi: 10.3389/fimmu.2026.1889571 (PMC13627372; doi:10.3389/fimmu.2026.1889571)
Supplement: Supplementary file 1 [file Table1.docx]

**Supplementary Tables**

**Supplementary Table 1 Intra-observer and Inter-observer Agreement (ICC) for Macroscopic Fractal Feature Extraction**

| **Imaging Feature** | **Intra-observer ICC (95% CI)** | **Inter-observer ICC (95% CI)** | **Robustness Status** |
| --- | --- | --- | --- |
| Structural Fractal Dimension (FD_MRI) | 0.934 (0.885–0.962) | 0.892 (0.814–0.938) | Highly Robust / Retained |
| Metabolic Fractal Dimension (FD_PET) | 0.915 (0.852–0.951) | 0.876 (0.785–0.929) | Highly Robust / Retained |

**Notes:** Cohort sampling: A subset of 50 patient scans was randomly selected from the training cohort for quality control and reproducibility evaluation. Intra-observer agreement was calculated by Radiologist A independently re-delineating the 3D-ROI and extracting features from the same batch of 50 images after a 1-month interval. Inter-observer agreement was calculated by comparing the initial feature extraction results independently generated by Radiologist A and Radiologist B in a double-blinded state. ICC values > 0.80 indicate excellent reliability. In this study, both FD_MRI and FD_PET demonstrated high geometric fidelity across decoupled multimodal extractions, meeting the pre-defined inclusion threshold (ICC > 0.80) for XGBoost modeling.

**Supplementary Table 2 Optimal Hyperparameters of the Multiscale XGBoost Model (IRJS) Determined via GridSearchCV**

| **Hyperparameter** | **Search Space** | **Final Selected Value** | **Parameter Description** |
| --- | --- | --- | --- |
| n_estimators | [50, 100, 150, 200, 300] | 150 | Maximum number of decision trees. |
| learning_rate (eta) | [0.01, 0.05, 0.1, 0.2] | 0.05 | Step size shrinkage used in update to prevent overfitting. |
| max_depth | [3, 4, 5, 6, 7] | 3 | Maximum depth of a tree; restricted to 3 to learn higher-order non-linear features. |
| min_child_weight | [1, 2, 3, 5] | 3 | Minimum sum of instance weight needed in a child. |
| subsample | [0.6, 0.7, 0.8, 0.9, 1.0] | 0.8 | Subsample ratio of the training instances. |
| colsample_bytree | [0.6, 0.7, 0.8, 0.9, 1.0] | 0.7 | Subsample ratio of columns when constructing each tree. |
| gamma | [0, 0.1, 0.2, 0.5] | 0.2 | Minimum loss reduction required to make a further partition. |
| scale_pos_weight | [1.0, 1.5, 2.1, 2.5, 3.0] | 2.1 | Balancing of positive and negative weights for class imbalance. |

**Notes:** GridSearchCV Strategy: Hyperparameter tuning was systematically executed utilizing a Grid Search algorithm integrated with 5-fold cross-validation (cv=5) exclusively within the discovery and training cohort (Center A, 2019-2024, N=150). Evaluation Metric: The optimization objective was the Area Under the Receiver Operating Characteristic Curve (ROC-AUC) to ensure maximal discriminatory power. Overfitting Prevention: Given the sample size relative to the multi-scale feature matrix, parameters enforcing strong regularization (e.g., lower max_depth, active subsampling, and positive gamma) were selected. The scale_pos_weight of 2.1 successfully countered intrinsic class imbalance, penalizing the misclassification of the minority "Immune-inflamed" phenotype without artificial data synthesis.

**Supplementary Table 3 Variance Inflation Factor (VIF) Collinearity Diagnostics for Features Included in the Multiscale Joint Model (IRJS)**

| **Variables** | **VIF Value** | **Tolerance** | **Collinearity Status** |
| --- | --- | --- | --- |
| Systemic Immune-Inflammation Index (SII) | 2.68 | 0.373 | No Multicollinearity |
| Neutrophil-to-Lymphocyte Ratio (NLR) | 2.45 | 0.408 | No Multicollinearity |
| Lactate Dehydrogenase (LDH) | 1.52 | 0.658 | No Multicollinearity |
| Structural Fractal Dimension (FD_MRI) | 1.36 | 0.735 | No Multicollinearity |
| Metabolic Fractal Dimension (FD_PET) | 1.28 | 0.781 | No Multicollinearity |
| *KRAS* Mutation Status | 1.15 | 0.870 | No Multicollinearity |
| MSI-H / dMMR Status | 1.04 | 0.962 | No Multicollinearity |

**Notes:** VIF measures the degree of multicollinearity in a set of regression variables. A VIF value of 1 indicates no correlation, while values between 1 and 5 suggest moderate correlation, generally acceptable in clinical machine learning models. In this study, a rigorous pre-modeling threshold of VIF < 3.5 was enforced to explicitly eliminate severe multicollinearity interference. This quality control ensures that each feature independently retains sufficient explained variance, satisfying the precondition for the post-hoc attribution mapping generated by the SHAP algorithm. Tolerance is calculated as 1/VIF.
**Supplementary Table 4 Diagnostic Performance Comparison Between XGBoost and Penalized Logistic Regression**

| **Cohorts and Models** | **AUC (95% CI)** | **Accuracy (%)** | **Sensitivity (%)** | **Specificity (%)** | **F1-Score** | **P-value (DeLong)*** |
| --- | --- | --- | --- | --- | --- | --- |
| **Training Cohort (n = 150)** |  |  |  |  |  |  |
| L1-Penalized LASSO Regression | 0.854 (0.80–0.90) | 79.3 | 77.1 | 80.4 | 0.782 | 0.012 |
| Joint Multiscale Model  (XGBoost / IRJS) | 0.921 (0.88–0.96) | 87.3 | 89.6 | 86.3 | 0.885 | Reference |
| **Temporal Validation Cohort (n = 45)** |  |  |  |  |  |  |
| L1-Penalized LASSO Regression | 0.825 (0.74–0.90) | 75.6 | 73.9 | 76.5 | 0.730 | 0.034 |
| Joint Multiscale Model  (XGBoost / IRJS) | 0.886 (0.80–0.97) | 82.2 | 87.5 | 79.3 | 0.848 | Reference |
| **External Validation Cohort (n = 75)** |  |  |  |  |  |  |
| L1-Penalized LASSO Regression | 0.798 (0.71–0.87) | 74.7 | 72.0 | 76.1 | 0.725 | 0.041 |
| Joint Multiscale Model  (XGBoost / IRJS) | 0.862 (0.79–0.94) | 82.6 | 80.0 | 78.8 | 0.817 | Reference |

**Notes:** Abbreviations: AUC, Area Under the Receiver Operating Characteristic Curve; CI, Confidence Interval; LASSO, Least Absolute Shrinkage and Selection Operator; IRJS, Immune-Radiomics Joint Score.

Model Specification: Both the L1-Penalized LASSO Regression and the XGBoost model were trained utilizing the exact identical set of core features (including FD_MRI, FD_PET, SII, LDH, and *KRAS* status) following Variance Inflation Factor (VIF) filtering. The LASSO model was internally optimized using 5-fold cross-validation to determine the optimal regularization penalty to enforce variable shrinkage and selection.

(*) Statistical Significance: The *P*-value was calculated using DeLong’s test for two correlated ROC curves, explicitly evaluating whether the non-linear, gradient-boosted tree architecture of XGBoost (IRJS, set as the statistical Reference) provided a significant incremental improvement in discriminatory capacity over the conventional linear mapping assumption inherent to the LASSO regression model across all spatiotemporal cohorts.
